# The Case of a 19‐Year‐Old Woman Presenting With Headache and Transient Loss of Consciousness

**DOI:** 10.1002/acn3.70338

**Published:** 2026-03-19

**Authors:** Yan Lin, Xiaoyu Zhou, Lin Shen, Kunqian Ji, Yuying Zhao, Chuanzhu Yan, Yiming Liu, Wei Li

**Affiliations:** ^1^ Department of Neurology Qilu Hospital, Shandong University Jinan China; ^2^ Shandong Key Laboratory of Mitochondrial Medicine and Rare Diseases, Research Institute of Neuromuscular and Neurodegenerative Diseases Qilu Hospital of Shandong University Jinan Shandong China; ^3^ Chengwu Public Hospital of Traditional Chinese Medicine Heze China

**Keywords:** autoimmunity, GFAP, inflammation

## 1 Summary of Case

**FIGURE 1:**
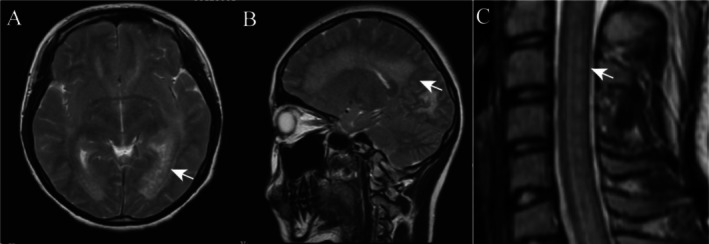
MRI brain w/wo before treatment, pre‐contrast. T2 weighted images (A–C) showed linear hypertensities adjacent to the fourth ventricle, bilateral lateral ventricles, cervical spine (indicated by arrows), and corona radiata and pons (not shown).

**FIGURE 2:**
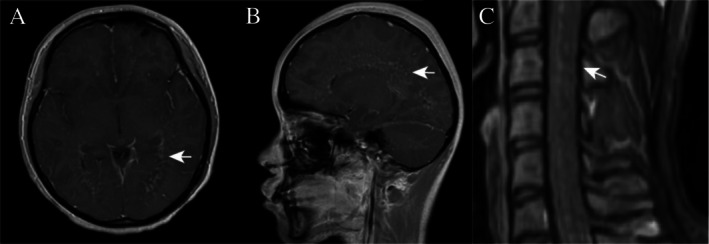
MRI brain w/wo before treatment, post‐contrast. T1 weighted images (A–C) showing patchy perivascular enhancement of the corona radiata area and enhancement in cervical spine.

A 19‐year‐old woman presented with intermittent fever, pulsatile temporal headaches, transient loss of consciousness, and blurred vision. Imaging showed multifocal patchy T1/T2 hyperintensities with perivascular enhancement in the periventricular regions and pons (Figure 1). CSF analysis revealed lymphocytic pleocytosis, markedly elevated protein and pressure. Marked improvement in both clinical manifestations and neuroimaging findings (Figure 2) was observed following high‐dose corticosteroid pulse therapy. This case highlights a rare but recognizable inflammatory encephalopathy with distinctive radiological and CSF features.

## 2 Diagnosis

Autoimmune glial fibrillary acidic protein astrocytopathy (GFAP‐A).

## 3 Take‐Home Points

Autoimmune GFAP astrocytopathy (GFAP‐A) should be considered in patients with subacute onset of meningoencephalitic symptoms and distinctive MRI features.

Definitive diagnosis of GFAP‐A relies on the detection of GFAP antibodies in the cerebrospinal fluid, in combination with characteristic clinical and radiologic findings.

High‐dose corticosteroids, often in combination with intravenous immunoglobulin (IVIG), constitute the first‐line therapy and typically lead to rapid clinical improvement.

Symmetrical, radial periventricular enhancement on MRI is a hallmark imaging feature of GFAP‐A and serves as a key diagnostic clue.

GFAP‐A may be associated with underlying malignancies; therefore, comprehensive tumor screening is recommended after diagnosis.

## Author Contributions

Yan Lin and Xiaoyu Zhou: clinical data collection; writing – original draft. These authors contributed equally to this work. Lin Shen: data collection; Kunqian Ji, Yuying Zhao, and Chuanzhu Yan: supervision; Yiming Liu and Wei Li: writing – review and editing.

## Funding

The authors have nothing to report.

## Conflicts of Interest

The authors declare no conflicts of interest.

## Data Availability

The data that support the findings of this study are available on request from the corresponding author due to privacy or ethical restrictions.
